# A compulsive act of excess water intake leading to hyponatraemia and rhabdomyolysis: a case report

**DOI:** 10.1186/s12245-019-0255-6

**Published:** 2019-11-14

**Authors:** Sudheera Fernando, Francisca Sivagnanam, Devarajan Rathish

**Affiliations:** 1 Medical Unit, National Hospital of Sri Lanka, Colombo, Sri Lanka; 2 Department of Pharmacology, Faculty of Medicine and Allied Sciences, Rajarata University of Sri Lanka, Anuradhapura, Sri Lanka

**Keywords:** Water intoxication, Hyponatraemia, Hypokalaemia, Rhabdomyolysis, Ureteric calculus

## Abstract

**Background:**

Primary polydipsia is commonly seen in patients with psychiatric illnesses. Excess water intake is also seen in patient with anorexia and anticholinergic medications. We report a patient who had hyponatraemia and rhabdomyolysis after consuming excess water for ureteric calculus.

**Case presentation:**

A healthy middle-aged male presented with an episode of generalized tonic-clonic seizure and reduced level of consciousness preceded by consumption of excess water. He was recently diagnosed to have a ureteric calculus and was advised to consume plenty of water. On examination, he was disoriented in place, person and time. Except for the generalized diminished reflexes, other neurological and systemic examinations were normal. He had severe hyponatraemia, mild hypokalaemia and myoglobulinuria. His serum creatinine phosphokinase and aspartate aminotransferase were markedly elevated. The diagnosis of rhabdomyolysis in the setting of acute water intoxication was made. Optimum fluid and electrolyte management achieved a dramatic recovery of consciousness, hyponatraemia and rhabdomyolysis.

**Discussion:**

The patient has had excess water intake due to a compulsive act in the background fear of ureteric calculus. Such act could lead to severe hyponatraemia and rhabdomyolysis. Therefore, future similar acts could be prevented by proper medical advice. Further, emergency physicians should be vigilant for rhabdomyolysis in patients with hyponatraemia or hypokalaemia.

## Background

Psychogenic polydipsia can induce hyponatraemia [1–5]. Hyponatraemia in turn could lead to cerebral oedema and subsequent neurological complications like seizure or coma. Moreover, hyponatraemia could cause rhabdomyolysis [6]. This is a serious complication which could be easily ignored. Therefore, polydipsia can rarely lead to rhabdomyolysis and prevention of which needs optimum vigilance [1–9]. We discuss the management and monitoring of a patient who had acute water intoxication induced hyponatraemia and rhabdomyolysis after being advised to consume water for his recently diagnosed ureteric calculus.

## Case presentation

### History and examination

A 41-year-old male was in good health until 2 weeks ago when he developed a left-sided renal colicky type pain and was diagnosed to have a left mid ureteric calculus by radiography. He was reassured and requested to consume plenty of water. Two days before, he had consumed excessive amount of water, about 10 l per day, and developed polyuria. During these 2 days, he has had fever, dark urine, generalized weakness and altered level of consciousness. On the day of admission, he developed a single episode of self-terminating generalized tonic-clonic seizure lasting for 5 min followed by reduced level of consciousness. There was no previous history or family history of adult-onset seizures. There were no features suggestive of psychiatric illness like low mood, reduced sleep or overtalkative behaviour. The patient was never transfused with blood or blood products. There was no recent travel history. He is married and has a 10-year-old child. He is an accountant by profession. The patient has had good income and family support. He occasionally consumed alcohol and smoked cigarette, but denied any drug abuse. He was not on any long-term medication. On examination, he was disoriented in place, person and time. The Glasgow coma scale (GCS) score was 9 on admission (motor—5, verbal—2, eye—2). Except for a temperature of 101 °F and the generalized diminished reflexes, other vital signs and cardiovascular, respiratory, abdominal and neurological examinations were normal. Table 1 outlines the timeline of this case report.

**Table 1:** Timeline of the case report

Time point	Clinical features	Investigations	Management
2 weeks prior to hospital admission	• Left-sided renal colicky type pain	• Radiography—left mid ureteric calculus	• Reassured and requested to consume plenty of water
2 days prior to hospital admission	• Consumed about 10 l of water per day and developed polyuria, fever, dark urine, generalized weakness and altered level of consciousness	• None	• None
On the day of hospital admission	• One episode of generalized tonic-clonic seizure • Disoriented in place, person and time • GCS—9 (motor—5, verbal—2, eye—2)	• Serum sodium—119 mmol/L • Serum potassium—2.9 mmol/L • Serum osmolality—242 mOsm/kg of water • Urine osmolality—195 mOsm/kg of water • Non-contrast CT brain—normal	• IV cefotaxime (2 g 6 hourly) • IV acyclovir (500 mg 8 hourly) • IV dexamethasone (2 mg 6 hourly) • IV 3% hypertonic NaCl single bolus (100 mL over 20 min) • IV KCl single infusion (40 mmol over 12 h) • Fluid input per hour = previous hours output + 20 mL
Day 3	• GCS—15 (motor—6, verbal—5, eye—4)	• Serum sodium—136 mmol/L • Serum potassium—3.8 mmol/L • Creatinine phosphokinase—10,000 U/L • Myoglobinuria—positive	• Fluid input per hour = previous hours output + 20 mL • IV antibiotics continued
Day 4	• Clinically stable	• AST—2316 U/L • ALT—463 U/L	• Fluid input per hour = previous hours output + 20 mL • IV antibiotics continued
Day 5	• Clinically stable	• Serum osmolality—342 mOsm/kg of water • Urine osmolality—869 mOsm/kg of water	• Fluid input per hour = previous hours output + 20 mL • IV antibiotics continued • IV dexamethasone omitted
Day 7	• Clinically stable	• AST—1383 U/L • ALT—488 U/L • Creatinine phosphokinase—54,841 U/L	• Normal fluid intake per day • IV antibiotics continued
Day 9	• Clinically stable	• AST—702 U/L • ALT—398 U/L • Creatinine phosphokinase—18,070 U/L	• Normal fluid intake per day • IV antibiotics continued
Day 11	• Clinically stable	• AST—240 U/L • ALT—232 U/L • Creatinine phosphokinase—1419 U/L	• Normal fluid intake per day • IV antibiotics omitted
Day 15	• Clinically stable	• AST—66 U/L • ALT—78 U/L • Creatinine phosphokinase—49 U/L	• Discharged with appropriate medical advice to prevent similar complications

### Differential diagnosis

This is a previously healthy male who presented with fever for 2 days complicated with generalized tonic-clonic seizure and reduced level of consciousness. In the acute setting, the first working diagnosis was meningoencephalitis. It could be due to viral or bacterial aetiology. Moreover, he had dark coloured urine; therefore, falciparum malaria which can led to cerebral malaria was also suspected. Collateral history from the patient’s family revealed an excessive water intake of about 10 l per day for 2 days which led to an additional differential diagnosis. One possibility is psychogenic polydipsia which is the most common cause for excessive water intake. However, there was no history of psychiatric illness or any long-term drug history which can lead to polydipsia. The reason for his excessive water intake was to get rid of the unpleasant pain due to the ureteric calculus.

### Investigation and management

On admission, he was found to have hypoosmolar (242 mOsm/kg of water), hyponatraemia (119 mmol/L), hypokalaemia (2.9 mmol/L), hypoosmolar urine (195 mOsm/kg of water), mild leucocytosis (12.7 × 10^3^ per microliter) with neutrophilia and myoglobinuria. His serum creatinine phosphokinase (10,000 U/L) was markedly elevated with a rise in aspartate aminotransferase (AST) (2316 U/L) and alanine aminotransferase (ALT) (463 U/L) levels. Renal functions were within normal ranges. Blood for malaria parasite was negative. Non-contrast computed tomography scan of the brain was normal. Electroencephalogram done on the 5th day revealed generalized, continuous, slow activity suggestive of diffuse cortical dysfunction. This could be due to an encephalopathy preceded by hyponatraemia.

Hyponatraemia-induced generalized tonic-clonic seizure, altered level of consciousness and rhabdomyolysis were diagnosed in our patient. His hyponatraemia was most probably due to excess water intake. The patient was given a bolus of 3% hypertonic saline (100 mL over 20 min) for symptomatic severe hyponatraemia [10]. The diagnosis of rhabdomyolysis was made because he had body aches, dark urine, elevated levels of creatine kinase and urinary myoglobin. His serum creatinine phosphokinase peaked to the highest level (54,841 U/L) by the 7th day. Also, the patient’s elevated AST and ALT could be due to the muscle damage. As there was rhabdomyolysis, hypokalaemia correction was done once (KCl 40 mmol over 12 h) and serum potassium level was assessed regularly. Further doses of potassium were not required. A strict fluid restriction was not followed as there was a high risk of developing acute renal failure due to rhabdomyolysis. The input per hour was decided by measuring the previous hours’ output and adding 20 mL for the first 5 days. He did not develop oliguria or features of acute renal failure. Considering the history of fever and seizure with altered level of consciousness, he was started on intravenous cefotaxime (2 g 6 hourly), intravenous acyclovir (500 mg 8 hourly) and intravenous dexamethasone (2 mg 6 hourly) after collecting blood and urine for culture. However, the patient’s family denied consent for lumbar puncture. Blood and urine culture revealed no growth, but the antibiotics were continued for a total of 10 days as we could not exclude central nervous system infection without a lumbar puncture. The magnetic resonance imaging of the brain was normal on the 11th day of admission.

### Outcome

GCS score improved gradually to 15, and serum sodium and potassium were normalized by the 3rd day of admission. The patient did not have any further episode of seizure. His fever settled, and serum and urine osmolality were normalized by the 5th day. He responded to the above management and did not develop any complications of rhabdomyolysis. On discharge, his serum creatinine phosphokinase (49 U/L), AST (66 U/L) and ALT (78 U/L) levels were near normal. He was discharged on the 15th day of admission with appropriate medical advice to prevent similar complications.

## Discussion

Cases of acute water intoxication leading to hyponatraemia and rhabdomyolysis were found in few reports of previous literature. The first such case was reported by Browne PM in 1979 where an elderly patient drank excess water to increase his urine output which he thought was low [11]. Another patient was reported in 1987 when a 64-year-old patient with major depression developed self-induced water intoxication associated with rhabdomyolysis [12]. Most of the other cases were reported in patients with psychiatric illness [1–8], while Putterman et al. reported on a patient who had acute voluntary water intoxication following exercise [9]. However, our patient had an acute voluntary water intoxication following a compulsive act for renal colicky type pain.

Hyponatraemia is the most common electrolyte abnormality in the hospitalized patients [13]. Severity of its presentation depends on how rapidly the condition develops and the degree of cerebral oedema. Symptoms of hyponatraemia include poor concentration, anorexia, headache, irritability, restlessness, nausea, vomiting, fatigue, disorientation, confusion, seizure and respiratory arrest [14, 15]. Too rapid correction of hyponatraemia could cause a rapid shift in plasma osmolality and lead to central or extra pontine myelinolysis [16]. Thus, in our patient, a gradual correction of hyponatraemia was achieved. Causes of hyponatraemia are divided according to the volume status (euvolaemic, hypovolaemic and hypervolaemic) [17]. As the patient had a euvolaemic status, possible cause for euvolaemic hyponatraemia in this patient could be primary polydipsia ahead of syndrome of inappropriate antidiuretic hormone secretion, hypothyroidism or reset osmostat [17]. Primary polydipsia is a well-known entity which is commonly seen in patients with psychiatric illness like schizophrenia or in patients who are on antipsychotic treatment [18]. However, our patient had excessive water intake that led to hyponatraemia due to a compulsive act of fear for ureteric calculus.

Polydipsia-induced hyponatraemia can rarely lead to rhabdomyolysis [1–9, 19]. Rhabdomyolysis is a rare but serious clinical entity, and the mechanism of its occurrence is unclear [6]. Moreover, potassium released from muscles during exercise can cause vasodilation and increase the blood flow to the muscle. Thus, hypokalaemia may induce rhabdomyolysis by reducing blood flow to muscles in response to exertion. In our patient, both hyponatraemia and hypokalaemia were noted which might have contributed for the development of rhabdomyolysis. Rhabdomyolysis is a condition characterized by muscle necrosis and the release of intracellular muscle constituents into the circulation. Classic triad of muscle pain, muscle weakness and dark coloured urine was not seen in all patients [20]. Above half of the patients with confirmed rhabdomyolysis do not exhibit the muscle symptoms [20]. The spectrum of disease ranges from asymptomatic elevations in serum muscle enzymes to life-threatening illness associated with acute kidney injury and electrolyte imbalances [21]. Management of rhabdomyolysis should include treatment of the cause and prevention of its complications such as acute kidney injury. For the prevention of acute kidney injury, early and aggressive fluid resuscitation is essential. But our patient had water intoxication and hyponatraemia; therefore, fluid resuscitation was done with less aggression.

Polydipsia-induced hyponatraemia is a rare cause of rhabdomyolysis. Emergency physicians should be vigilant of this potential complication. One of the key points to learn from this case is that the physician should consider checking a serum creatinine phosphokinase level in patients presenting with hyponatraemia or hypokalaemia. Future studies need to explore the mechanism by which hyponatraemia could cause rhabdomyolysis. The patient has had excess water intake due to a compulsive act leading to severe hyponatraemia and rhabdomyolysis. Therefore, compulsive act of excess water intake should be prevented by individualized medical advice. Further, emergency physicians should keep in mind that hyponatraemia or hypokalaemia can be associated with rhabdomyolysis.

## Data Availability

All data generated or analysed during this study are included in this published article.

## Abbreviations

ALT: Alanine aminotransferase
AST: Aspartate aminotransferase
GCS: Glasgow coma scale
